# Long term toxicity and prognostic factors of radiation therapy for secreting and non-secreting pituitary adenomas

**DOI:** 10.1186/1748-717X-8-18

**Published:** 2013-01-23

**Authors:** Stefan Rieken, Daniel Habermehl, Thomas Welzel, Angela Mohr, Katja Lindel, Jürgen Debus, Stephanie E Combs

**Affiliations:** 1 Department of Radiation Oncology, University Hospital of Heidelberg, Im Neuenheimer Feld 400, 69120, Heidelberg, Germany; 2 Neuro-Radiation Oncology Research Group, Department of Radiation Oncology, University of Heidelberg, Im Neuenheimer Feld 400, 69120, Heidelberg, Germany

**Keywords:** Pituitary, Adenoma, Secreting, Stereotactic, Endocrine, Toxicity

## Abstract

**Background:**

Radiotherapy is controversially discussed in the management of benign disorders for fear of late sequelae such as tumor induction. This study was initiated to investigate long-term toxicity, treatment outcome and prognostic factors after radiotherapy (RT) in patients with pituitary adenomas.

**Methods:**

92 patients with pituitary adenomas were included in this analysis. RT was conducted using either 3D conformal (16%) or fractionated stereotactic techniques (83%) in a postoperative adjuvant setting (16%), as second-line treatment for recurring tumors (78%) or as primary treatment (6%). Postoperatively, RT was offered to patients with residual tumor tissue or in case of locally extensive adenomas, in whom early recurrence was deemed likely. Patients were followed for a median time of 152.5 months, and analysed for overall and local progression-free survival (OS and LPFS). Multiple factors were analysed for prognostic impact. Patients were contacted with an institutional questionnaire about qualiy of life (QOL). Statistical analysis was performed using the log-rank test and the Kaplan-Meier method using a software tool (SPSS 19.0).

**Results:**

Median follow-up was 152.5 months. Before treatment, 2% of all patients were diagnosed with adenoma-related hypopituitarism. Following surgery, 68% suffered from new pituitary deficits. RT was associated with mild toxicity, including visual deficits (5.4%) and hypopituitarism (10.9%). In particular, no radiation-induced brain necrosis or malignancy was observed. QOL was reported to be stable or improved in 92% of all patients, and RT was perceived to not compromise but increase QOL in the vast majority of patients (95%). OS after RT was 93.3% and 61.0% at 120 and 240 months. LPFS following RT was 90.4 and 75.5% at 120 and 240 months. Early initiation of RT after surgery instead of reserving it for recurring adenomas predisposed for improved outcome.

**Conclusions:**

RT for pituitary adenomas is safe and and self-reported QOL is stable or improved by almost all patients. Hypopituitarism rates are low. Local control appears improved in patients irradiated postoperatively over those undergoing RT for previously resected recurrent tumors.

## Introduction

Pituitary adenomas are benign tumors representing 10 – 15% of primary intracranial tumors. Depending upon their histopathology, they are inactive or hormone-secreting lesions [1,2]. They are frequent causes of pituitary dysfunction comprising both suppression-related hypopituitarism, but also adenoma-derived hypersecretion [3]. Besides endocrinological morbidity, pituitary adenomas often lead to visual impairment related to both optic nerve affection with reduced visual fields and acuity, but also disturbed oculomotor function – especially in cases of cavernous sinus infiltration [4]. It has been discussed controversially whether RT should be delivered after surgery, which is regarded as the preferential initial treatment. Additionally, substantial discussion has focussed on which risk factors can be identified arguing for early adjuvant treatment [4-6].

In general, indication for RT is set for recurrent tumors, for atypical histology, or for persistent hormone secretion. Cranial RT for pituitary adenomas, but also for target volumes in close proximity to the sella has repeatedly been reported to be a cause of hypopituitarism [7-9]. Varying intervals between RT and decline in hormone levels have been published, ranging from one to more than ten years [10,11]. Also, distinct threshold doses for the various hormone-secreting cells have been described, resulting in characteristic clinical courses, commonly starting with loss of growth hormone [9]. Taking further potential sequelae such as visual deficits [12], cerebral radiation injury [13] and tumor induction [1,14] into consideration, the role of RT for benign lesions of the pituitary gland has repeatedly been challenged. Moreover, controversial data on when to irradiate (primary vs. postoperative vs. salvage), whom to irradiate (secreting vs. non-secreting) and how to irradiate (fractionated stereotactic RT vs. radiosurgery vs. intensity modulated RT) have occupied scientists, clinicians and patients [5,15-17]. Several authors have addressed short- and medium-term outcome in irradiated pituitary adenoma patients [18,19], however little data is available on long results and toxicity.

We previously analysed short-term outcome with conventional RT and fractionated stereotactic RT for pituitary adenomas [2,20,21]. In the present report, we summarized our long-term experiences in the management of pituitary adenomas after a follow-up of more than 10 years in 92 patients.

## Patients and methods

Between 1984 and 2010, 92 patients with pituitary adenomas were treated at the University hospital in Heidelberg, Germany. All data were collected retrospectively and in accordance with institutional ethical policies. Additionally, patients were contacted to update their charts on current hormone replacement medication, QOL and potential additional therapies. Median follow-up time since first diagnosis was 152.5 months (range 9 – 548 months) and 99 months (range 1 – 310 months) since RT.

### Patient characteristics

Patient characteristics are summarized in Table 1.

**Table 1:** Summary of patient characteristics

**Patient characteristics**			
**biometric**		(years)	(range)
	median age	51	10 - 88
		(n)	(%)
	female	33	36
	male	59	64
**Initial symtoms**		(n)	(%)
	asymptomatic	18	20
	pituitary subfunction	2	2
	hormonal excess	29	32
	reduced visual field / acuity	29	32
	cerebral symptoms (headache, nausea, neuropsycholgical)	14	15
**histology**		(n)	(%)
	non-secreting	55	60
	GH-secreting	22	24
	Prolactine-secreting	8	9
	ACTH-secreting	6	7
	TSH-secreting	1	1
		(n)	(%)
**surgery**		any	87
	complete	35	38
	incomplete	52	57
		(n)	(%)
**re-surgery**		any	48
	complete	8	9
	incomplete	40	44
		(n)	(%)
**radiotherapy**		3D conformal	15
	FSRT	76	83
	IMRT	1	1
		(Gy)	(range)
	median single dose	1.8	1.8 – 2.0
	median total dose	52.2	48.6 – 58.0
		(n)	(%)
	primary definitive RT	5	5
	postoperative RT	15	16
	RT for recurrent tumors	72	78

In 31 patients, endocrine symptoms were present at first diagnosis, including 29 patients with signs of hypersecretion and 2 with suppression of pituitary function. Impairment of the optic nerve was present in 28 patients (30%) at first diagnosis, while disturbed oculomotor function was diagnosed in 6 patients (7%).

### Surgery and histopathological findings

Surgery was chosen as first treatment in 87 patients (95%). Only 5 patients with inoperable macroadenomas underwent primary RT. Extent of surgery was assessed by the surgeon. Surgery was deemed complete in 35 patients (40% of 87). Macroscopic tumor residues were described in 52 patients (60% of 87). In recurrent patients, re-surgery was performed in 48 patients (55% of 87) and was complete in 8 patients (17% of 48). Histological and blood hormone analysis identified 37 patients with secreting adenomas (40%), including 22 somatotropinomas (59%), 8 prolactinomas (2%), 6 corticotropinomas (16%), and one thyrotropinoma (3%). Non-functioning adenomas were diagnosed in 55 patients (60%).

### Radiotherapy

RT was carried out as fractionated stereotactitc irradiation in the majority of patients (n = 76, 83%). Before introduction of stereotactic methods in the mid 1990s, 15 patients were treated with 3D conformal techniques (16%). For reason of extensive cavernous sinus infiltration, 1 patient (1%) was treated with an IMRT treatment plan. Treatment was planned based upon CT and MRI images. Target volumes encompassed any visible tumor adding a safety margin of 2 to 3 millimeters. Median planning target volume was 25.6 milliliters. Median total dose was 52.2 Gy with median single doses of 1.8 Gy.

### Follow-up

Patients were seen 8 weeks after completion of irraditiation and yearly hereafter. We recommended testing of both visual acuity and field and blood chemistry analysis of hormonal state 6 and 12 months following RT. Most patients were monitored annually by their treating physicians. Hypopituitarism was defined as requirement for new hormonal replacement therapy. Imaging diagnosis was perfomred using MRI in all patients since the mid-90s. It was routinely included in the pretreatment planning procedure and additionally performed during follow-up with no fixed intervals, but at the discretion of the treating endocrinologists. Adenoma recurrence was diagnosed in case of new diagnosed tumor formation in cross sectional imaging or in case of re-increasing hormone levels. During this analysis, patients were recently contacted for updated information on present hormone replacement treatment. Also, they were asked about alterations of general QOL following initial diagnosis and treatment and whether or not QOL was changed by RT.

### Statistical analysis

OS was calculated from the date of primary diagnosis until the last date of follow-up or death. LPFS since first diagnosis and LPFS since RT were calculated in months from the date of initial diagnosis until the first imaging or serological-endocrine diagnosis of tumor relapse and from the date of the first RT fraction until the first imaging or serological-endocrine diagnosis of tumor relapse, respectively. Results were displayed with the Kaplan-Meier method. Survival curves were compared between groups by the log-rank test using a software tool (SPSS 19.0).

## Results

### Toxicity

RT was conducted without interruptions ≥ 4 days in all patients. No severe early or late treatment-related side effects were observed, including cerebral hemorrhages or brain necroses. At the time of initial diagnosis and prior to RT and surgery, 29 patients (31.5%) presented with low visual acuity or visual field deficits (31.5%). After surgical intervention, 14 new visual impairments were reported (16.1%). Of all patients at risk of visual side effects due to prior impairment, 5 patients complained about new symptoms following RT (5.4%), mostly comprising limited visual field deficits. There was no complete amaurosis following RT. There were no secondary malignancies. Five patients suffered from cancer during follow-up, however, none of them related to cranial RT fields (2 × prostate, 1 × gastric, 1 × lung, 1 × breast cancer).

Up-front tumor-related hypopituitarism was diagnosed in 2 patients (2.2%). Following surgical resection, 59 of 87 patients developed new pituitary deficit (68%). RT caused new deficits in 10 patients (10.9%), including thyreotrope (50%), somatotrope (40%), corticotrope (60%), and gonadotrope (20%) imbalances. All patients had received surgery prior to RT, and had also received partial hormone replacement therapy for postoperative deficits, indicative of preexisting postoperative damage of the pituitary gland. In reverse, no patient undergoing primary definitive RT for pituitary adenoma developed a new pituitary deficit. At the time of data collection, 71 patients were on hormone replacement therapy including mineralocorticotrope (80.6%), thyroxine (n = 52; 73.2%), testosterone (n = 46, 64.8%) and somatotrope (n = 16; 22.5%) substitution. Chronic administration of vasopressin was necessary in 5 patients (5.4%).

Patients reported high QOL following initial diagnosis and treatment with 24% stating improved and 68% stating stable QOL. Only 7% reported reduced QOL due to visual impairments in one and complicated or imbalanced hormonal replacement in two thirds. RT was reported to have improved or not affected QOL in 95% of all patients (17% and 78%). Reduction in QOL due to chronic toxicity was perceived by only 5%.

### Survival and prognostic factors

Median follow-up since the initial diagnosis of adenoma was 152.5 months (range 9 – 548 months). Most patients underwent RT for recurrent disease, therefore causing a median latency of 39 months between initial diagnosis and initiation of RT (range 1 – 404 months). After RT, median follow-up was 99 months (range 1 – 310 months).

**Figure 1:**
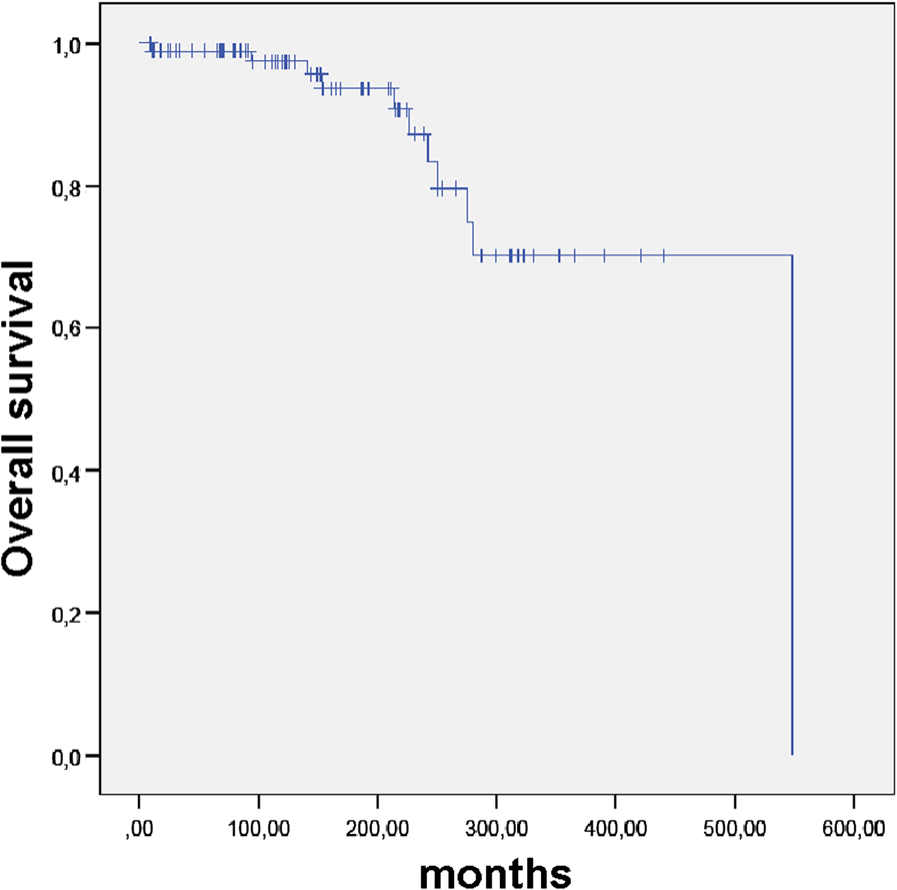
OS in 92 patients with pituitary adenomas after initial diagnosis.

**Figure 2:**
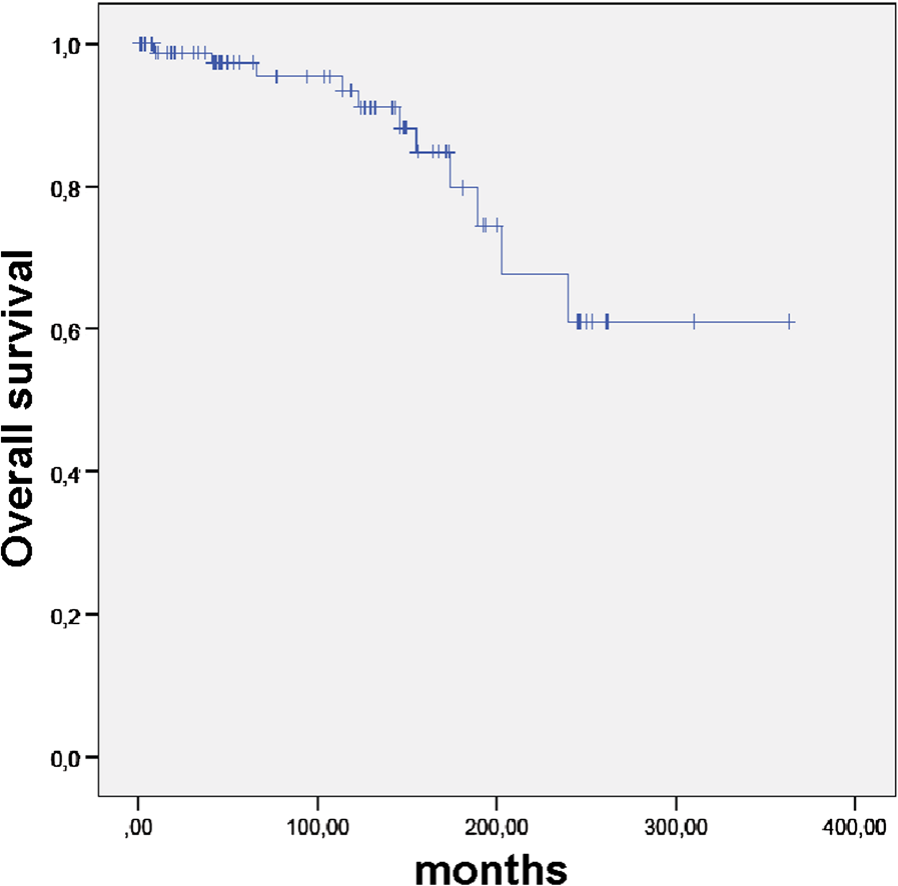
OS in 92 patients with pituitary adenomas after RT.

OS since first diagnosis was 98.9%, 97.4%, 87.2% and 70.2% at 12, 120, 240, and 360 months (Figure 1). OS after RT was 96.6%, 93.3%, 61.0%, and 61.0% at 12, 120, 240, and 360 months (Figure 2). Extent of surgery did not influence OS since initial diagnosis or since RT (p = ns).

Concerning endocrinological characteristics, OS was not influenced by hormone secretion (yes vs. no, p = 0.421). OS after 5, 10 and 20 years was 100, 96, and 81% in patients with functioning adenomas and 98, 98 and 90% in those with non-functioning adenomas. Patients with functioning adenomas had 5-, 10-, and 20-year LPFS rates of 87%, 81%, and 81% as opposed to 100, 100, and 74% in non-functioning adenomas. OS and LPFS were not affected by postoperative oor radiogenic hormonal deficits (yes vs. no; p = 0.445 and p = 0.863). Also, the need of hormone substitution therapy following treatment did not impair OS rates (p = 0.836). Of 92 patients, 31 were initially diagnosed with pituitary adenomas for reason of endocrine symptoms, including hypersecretion (n = 29) and tumor-related hypopituitarism (n = 2).

**Figure 3:**
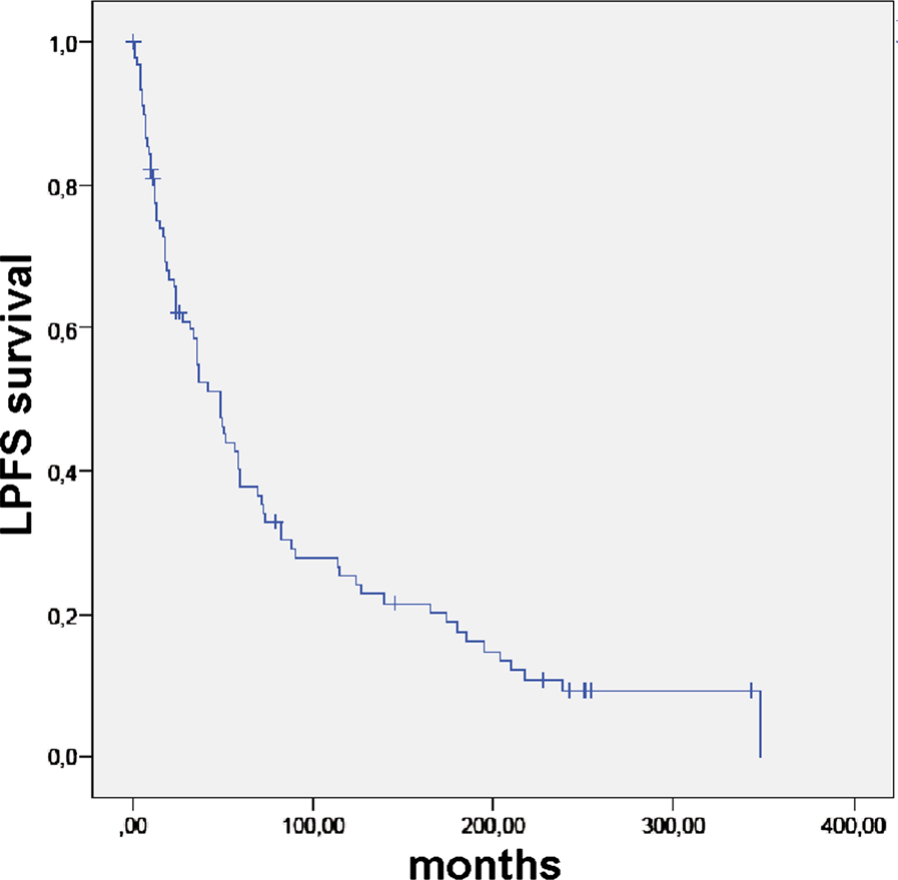
LPFS in 92 patients with pituitary adenomas after initial surgery.

**Figure 4:**
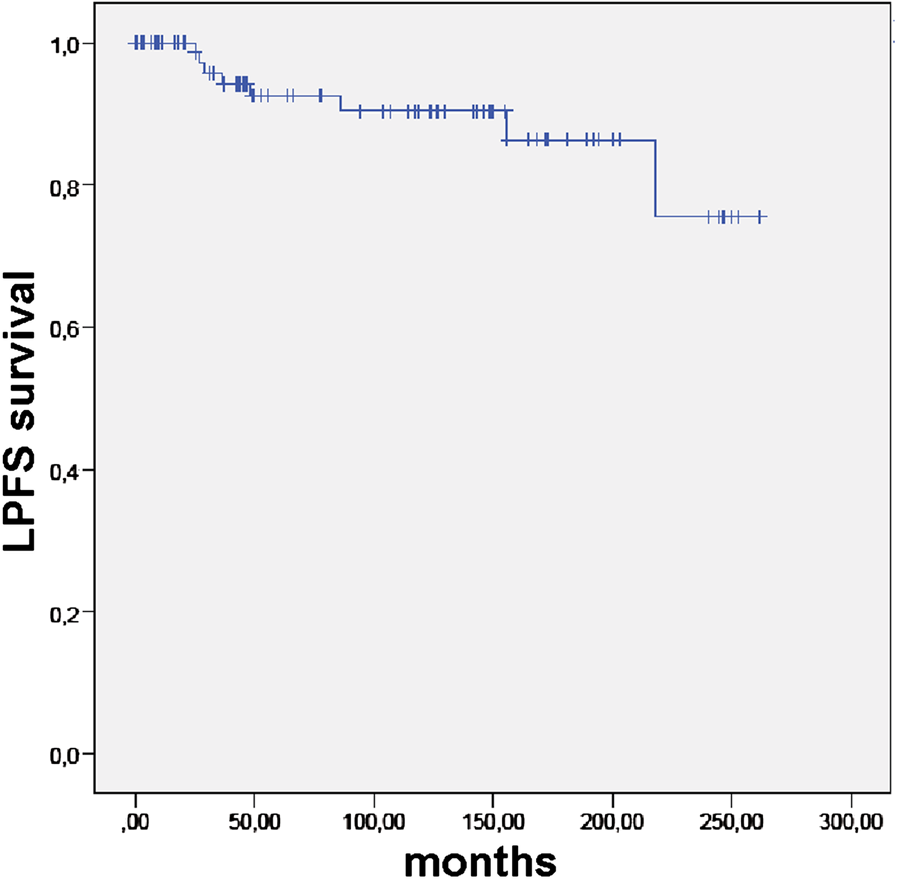
LPFS in 92 patients with pituitary adenomas after RT.

Most patients underwent RT for recurrent pituitary adenoma. Surgery for newly diagnosed pituitary tumor was chosen in 87 patients (94.6%). In this highly selected cohort with recurrent and locally advanced patients, LPFS since initial diagnosis and primary surgery was 80.9, 37.8, 25.3, and 9.2% after 12, 60, 120, and 240 months (Figure 3). It was not improved by complete vs. incomplete resection (p = 0.891). LPFS since RT was 100.0, 92.5, 90.4, and 75.5% after 12, 60, 120, and 240 months (Figure 4). LPFS following RT was not influenced by hormone secretion (secreting vs. non-secreting; p = 0.294), sex (male vs. female; p = 0.71), surgery (yes vs. no; p = 0.75), extent of resection (complete vs. incomplete; p = 0.45).

**Figure 5:**
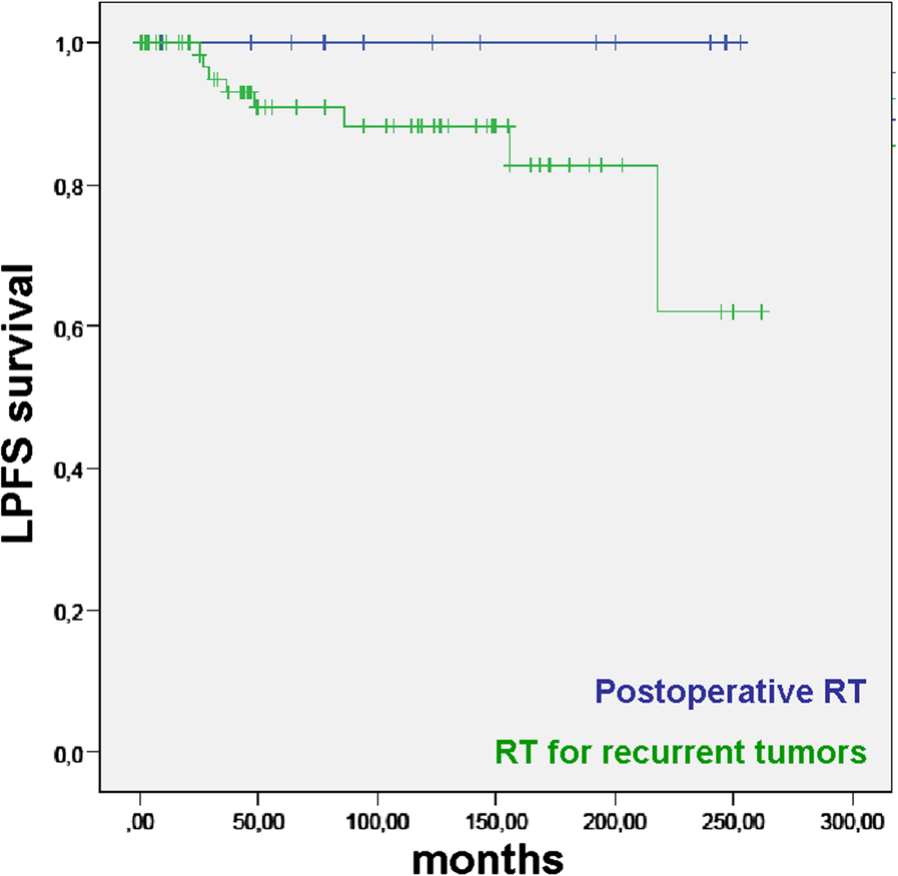
**LPFS in 87 patients treated with pituitary RT either postoperatively (blue line) or for relapsing adenomas (green line).** There is a strong trend towards improved local control in patients undergoing postoperative RT (p = 0.098/ns).

We observed a trend towards improved LPFS in those patients who received RT in a postoperative or primary setting instead of reserving it for patients with recurrent disease (p = 0.098/ns; Figure 5). There was no relapse in patients with primary or postoperative RT, whereas local control in patients treated for local recurrence was 100.0, 90.8, 88.2, and 62.0% after 12, 60, 120, and 240 months. Due to the small number of patients treated early after diagnosis (n = 18), statistical significance was not, yet, reached (p = 0.098).

### Patterns of relapse

Seven patients suffered from relapse after RT (7.6%). Median latency between RT and relapse was 36 months (range 27 – 156 months). Recurring adenomas were secreting in 4 patients (57.1%) and non-secreting in 3 patients (42.9%). In 5 patients, morphological tumor mass was present and detected through cross-sectional imaging, while in 2 patients with growth hormone secreting tumors, relapse was diagnosed based on blood hormone analysis with no macroscopic tumor tissue visible. Salvage treatments comprised reirradiation in 2 patients, resurgery in 3 patients, and sole pharmacological therapy in 2 patients.

There was no relapse in the 5 patients who received sole RT without prior surgical intervention. Also, no recurrence was diagnosed in patients undergoing surgery followed by early postoperative RT.

## Discussion

In the present manuscript, we analysed treatment outcome and toxicity in 92 patients with pituitary adenomas: We found RT to be safe and associated with little toxicity in primary, adjuvant and second-line line treatment regimes. Local control of disease was high with long patient survival. RT as a postoperative treatment option during first-line treatment appeared superior to reserving it for relapsing patients.

Previouly, our group had reported short-term outcome after conventional RT treated from 1972 to 1991 with promising outcome [21]. With the advent of high-precision techniques, these were implemented for benign lesions, and early outcome for secreting and non-secreting pituitary adenomas has been evaluated [2,20]. For benign lesions, however, long-term local control, QOL and chronic side effects are of utmost importance for the assessment of efficacy. Therefore, the present analysis has set one focus on these issues. Improved QOL was reported by 24% and stabilization of QOL was noted by 68% of all patients. Despite well-known risks for critical visual and endocrine functions, RT was reported to have exerted negative impact on QOL in only 5%, while 95% of all patients assessed RT to have secured or enhanced their QOL. Acute and chronic toxicity was very moderate in the current patient cohort, and no treatment-interrupting sequelae occurred. One third of all patients had visual deficits at the time of the initial diagnosis. Surgery caused additional visual symptoms in 16%, whereas RT was associated with new visual deficits in only 5% of all treated patients. Oruckaptan *et al.* reported on 684 patients, who were surgically treated for pituitary adenomas, and, too, found visual dysfunction to be the predominant symptom leading to hospitalization in 39 to 62% of all patients. Even though, in this cohort only few patients suffered from additional postoperative visual dysfunction, 17.5% of all patients required reoperation, 10% suffered from surgical complications and 1.6% died perioperatively [4]. This emphasizes that despite being routinely performed, surgery for pituitary tumors remains a perilous operative intervention.

No secondary cancer was observed. We consider this finding to be of great importance, since physicians may be hesitant to conduct RT for benign neoplasms in critical locations. One main concern against RT for benign lesions is the risk for long-term secondary malignancies, especially when treating younger patients. However, only few data is available supporting this concern. Isobe *et al*. described one out of 75 patients to suffer from suprasellar germinoma 45 months after RT [1]. Our work contributes new insight into this question since follow-up times are fairly long, confirming that secondary malignancies are no primary concern in the treatment of pituitary adenoma patients.

Pituitary dysfunction may cause severe medical conditions such as dysregulation of blood pressure or glucose metabolism, and is commonly associated with significantly reduced QOL, e.g. related to infertiliy or corticoid-associated stress adjustment disorders. Only 5% of all patients who answered the questionnaire reported reduced QOL related to RT, which supports data previously published by van Beek *et al*., who found no significant difference of QOL or cognition in patients undergoing postoperative irradiation vs. those who underwent surgery alone [22]. Following neurosurgical transsphenoidal resection, two of three patients developed a new pituitary deficit requiring pharmacological replacement. New pituitary deficits following RT were diagnosed in 11% of our patients, but all of these patients had undergone prior surgery and were on hormone replacement therapy for postoperative partial hypopituitarism. Reduced levels of TSH and ACTH were the predominant side effects of RT. Prior reports have identified the somatotropic axis to be most vulnerable to radiation damage [9]. However, since there was no routine monitoring of GH levels and since deficits were defined by pharmacological replacement, radiogenic damage to the somatotropic axis may be disguised by the majority of adult patients not being treated for asymptomatic growth hormone deprivation. Interestingly, radiogenic hypopituitarism was detected more frequently in patients who underwent irradiation for recurrent tumors than in those who were subjected to immediate postoperative RT.

This is the first report on a large cohort undergoing both primary RT, but also treatment of recurrent tumors with a long median follow-up of more than 8 years, including 16 patients with a follow-up of >15 years and 9 patients who were followed for more than 20 years after completion of RT. Weber *et al.* described 27 patients undergoing either adjuvant or radical RT for pituitary adenomas with a median follow-up of 6 years and found local control rates above 95% at 5 years [6]. Sun *et al.* reported on 33 patients, in whom local control rates of 94% were achieved at 36 months [3]. In our cohort, 10-year-local control rates were 90.4%, therefore supporting previously published data on high control rates in these patients. However, at 20 years after RT, local control had declined to 75.5%, indicating that long-term follow-up is mandatory in pituitary adenoma patients. Local control was not affected by patient sex and by hormone secretion. Langsenlehner *et al*. showed inferior local control of non-secreting adenomas [23]. This may be due to differing dose regimes with higher doses delivered to functioning adenomas. Also, various authors have demonstrated differential response of adenomas secreting different hormones, with somatotropinomas predisposing for improved response [1] and prolactinomas being associated with inferior outcome [4]. In this cohort, hormone secretion did not impact survival rates, and there was no difference in dose delivery between functioning and non-functioning adenoma patients (average dose: 51.4 vs. 52.2 Gy). All patients received doses >45 Gy, which are known to render control rates >90% [21]. The role of photon RT has been challenged, and previous authors have imputed inefficiency to postoperative RT. One of the largest cohorts supporting this hypothesis, however, had received median doses of 44.0 Gy. These doses are known to be ineffective in pituitary adenoma treatment [4].

Neurosurgical resection represents the standard treatment in newly diagnosed pituitary adenomas. In the group, reported here, neither extent of resection – as assessed by either cross-sectional imaging or surgical reports – nor surgery in general improved local control. This finding however is biased by selection with the majority of the patients reported here suffering from adenoma recurrences following surgery, not taking into account that many patients are considered cured following resection. We found that our patients, who represent those with a rather inferior prognosis due to either incomplete resection of extensive tumors or because of relapse after surgery, developed tumor recurrences after median latencies of 36 months with patients relapsing as late as 29 years after initial surgery. This finding emphasizes the need of regular long term follow-up examinations. This is impaired by the fact that until the mid 90s no 3D cross sectional and contrast agent-enhanced imaging was avaible to make residual disease visible and susceptible to further treatment. In our cohort, the majority of patients did not receive immediate post-operative imaging to control extent of resection.

Early postoperative RT was commonly indicated in case of incomplete resection mostly based upon the surgeons intraoperative assessments. We observed improved local control rates in these patients when comparing them to those only irradiated for tumor recurrences. Though statistically not yet significant, we consider this finding to be relevant, because physicians and patients may be hesitant to conduct early postoperative RT for fear of supraadditional toxicity or for keeping it as a salvage treatment in case of relapse. Knowing that local control is superior while toxicity remains unaltered if not benefical over patients treated for recurring tumors, we recommend that in case of likely relapse, such as in incomplete resected or locally advanced tumors, immediate postoperative RT can improve tumor control without serious sequelae.

We are aware of the limitations of our study: Comparing early postoperative RT with RT for recurrent RT in terms of improved LPFS, carries the risk of selection bias with possibly the more aggressive adenomas being included in the later group. Also, we acknowledge that no validated questionnaire was used to gather patient-reported data on QOL. However, we still consider the notion of generally improved QOL an important finding.

We conclude that RT for both functioning and non-functioning pituitary adenomas is safe and effective. It yields high local control rates with little neoplastic, cerebral, visual and endocrinological toxicity. QOL is not lowered by RT. In cases of inoperability, radical RT offers an effective and safe treatment modality.

## Abbreviations

ACTH: Adrenocorticotrope hormone; GH: Growth hormone; LPFS: Local progression-free surival; OS: Overall survival; QOL: Quality of life; RT: Radiotherapy; TSH: Thyroid stimulating hormone.

## Competing interests

There are no conflicts of interest to declare.

## Authors’ contributions

SR performed clinical analyses, assisted in patient treatment and wrote the manuscript. DH, AM, and KL helped to analyze patient data and organized follow-up examinations. TW helped with acquisition and interpretation of planning and follow-up imaging analyses. JD approved treatment plans, supervised patient treatment and financed the study. SC approved treatment plans, supervised patient treatment and helped to finalize the manuscript. All authors read and approved the current manuscript.
